# St Gallen molecular subtypes in primary breast cancer and matched lymph node metastases - aspects on distribution and prognosis for patients with luminal A tumours: results from a prospective randomised trial

**DOI:** 10.1186/1471-2407-13-558

**Published:** 2013-11-25

**Authors:** Anna-Karin Falck, Mårten Fernö, Pär-Ola Bendahl, Lisa Rydén

**Affiliations:** 1 Department of Surgery, Clinical Sciences, Lund University, Lund SE-22185, Sweden; 2 Department of Surgery, Hospital of Helsingborg, Helsingborg SE-251 87, Sweden; 3 Department of Oncology, Clinical Sciences, Lund University, Lund SE- 22185, Sweden; 4 Department of Surgery, Skåne University Hospital, Lund SE-22185, Sweden

**Keywords:** Breast cancer, Luminal A, Lymph node metastases, Molecular subtypes, Prognosis, Tumour progression

## Abstract

**Background:**

The St Gallen surrogate molecular subtype definitions classify the oestrogen (ER) positive breast cancer into the luminal A and luminal B subtypes according to proliferation rate and/or expression of human epidermal growth factor receptor 2 (HER2) with differences in prognosis and chemo-responsiveness. Primary tumours and lymph node metastases might represent different malignant clones, but in the clinical setting only the biomarker profile of the primary tumour is used for selection of adjuvant systemic treatment. The present study aimed to classify primary breast tumours and matched lymph node metastases into luminal A, luminal B, HER2-positive and triple-negative subtypes and compare the distributions.

**Methods:**

Eighty-five patients with available tumour tissue from both locations were classified. The distribution of molecular subtypes in primary tumours and corresponding lymph node metastases were compared, and related to 5-year distant disease-free survival (DDFS).

**Results:**

The St Gallen molecular subtypes were discordant between primary tumours and matched lymph node metastases in 11% of the patients (*p* = 0.06). The luminal A subtype in the primary tumour shifted to a subtype with a worse prognostic profile in the lymph node metastases in 7 of 45 cases (16%) whereas no shift in the opposite direction was observed (0/38) (*p* = 0.02). All subtypes had an increased hazard for developing distant metastasis during the first 5 years after diagnosis in both primary breast tumours and matched lymph node metastases, compared with the luminal A subtype.

**Conclusion:**

The classification according to the St Gallen molecular subtypes in primary tumours and matched lymph node metastases, implicates a shift to a more aggressive subtype in synchronous lymph node metastases compared to the primary breast tumour. The selection of systemic adjuvant therapy might benefit from taking the molecular subtypes in the metastatic node into account.

## Background

Breast cancer is a heterogeneous disease with variations in the biological profile and subsequent clinical prognosis. Prognostic information for the individual patient is based on the analysis of biological markers in the primary tumour including oestrogen receptor (ER), progesterone receptor (PR), human epidermal growth factor receptor 2 (HER2) and Ki67, together with age, tumour size, histological grade and lymph node engagement [1]. However, the clinical outcome varies despite identical biomarker profiles and stages: 20% of patients with node-negative breast cancer disease will have a recurrence and more than 30% of patients with lymph node metastases will remain disease-free [2,3]. Accordingly, a more precise prognostic tool is needed to identify patients who would benefit from adjuvant therapy as well as patients for which adjuvant therapy can be safely omitted.

Microarray-based gene expression studies [4,5] and subsequent immunohistochemical studies [6-9] have shown that further prognostic and predictive information can be gained by combining biological markers in the primary tumour rather than assessing them individually [6-8]. In 2011, the St Gallen International Breast Cancer Conference suggested a surrogate definition of intrinsic subtypes of breast cancer: luminal A (ER + and/or PR+, Ki67 low and HER2-), luminal B (ER + and/or PR+, Ki67 high and/or HER2+), HER2-positive (ER-, PR- and HER2+) and triple negative (ER-, PR-, HER2-) [10]. The classification has highlighted the heterogeneity of ER positive tumours in terms of prognosis. The luminal A subtype has a favourable prognosis compared to the luminal B subtype and the systemic therapy advocated for the patients with luminal A tumours is generally restricted to endocrine therapy. The luminal B subtype has a high proliferation rate and/or a high histological grade and systemic treatment with chemotherapy followed by endocrine therapy is recommended [10,11].

Selection of adjuvant systemic therapy is based on analysis of routinely used biomarkers in the primary tumour assuming that tumour biological markers are stable throughout tumour progression. Studies of paired samples of primary tumours and their metastatic lymph nodes and/or distant metastases suggest that tumour receptor status may be discordant in a fraction of patients [12-14] with influence on prognosis [13,15] proposing a more aggressive phenotype in the metastases in patients with disseminated disease. In a recent study, change of therapy according to biomarker expression in the metastatic site improved prognosis in the affected patients [16], stressing the clinical benefit of a biopsy of the recurrence as well as tailoring of therapy according to the biomarker profile in the metastatic location.

Analysis of individual biomarker expression (ER, PR, Ki67 and HER2) in primary breast cancer and synchronous lymph node metastases has shown that there is a small fraction of discordant cases but the prognostic implication for the individual patient is not settled [14,17-19]. Previous studies of biomarkers in synchronous lymph node metastases and asynchronous metastatic locations have focused on individual markers and lack information on the distribution of the St Gallen molecular subtypes. The present study aimed to investigate whether classification into luminal A, luminal B, HER2-positive and triple-negative subtypes provides information beyond that of the individual analyses of ER, PR, HER2 and Ki67 when comparing the inherence between the primary tumour and matched lymph node metastases in terms of distribution and prognosis. The St Gallen Guidelines from 2011 highlights the heterogeneity of ER positive disease with clear implications for selection of systemic adjuvant therapy and the present study addresses if analyses of the distribution of the intrinsic subtypes in synchronous metastatic lymph nodes can have therapeutic implications in addition to analyses of the primary tumour.

## Results

### St Gallen molecular subtype classification in the primary tumour and corresponding lymph node metastases

Patient and primary tumour characteristics are summarised in Table 1. In 9/85 cases (11%) the molecular subtype classification was discordant between the primary tumour and the lymph node metastasis (Table 2). The asymmetric pattern of the observed discordances indicates that the shift is non-random (*p* = 0.06, McNemar-Bowker test of symmetry). Moreover, 16% (7/45) of the cases which were luminal A in the primary tumour shifted to a subtype with a worse prognosis according to the lymph node metastases, whereas not a single shift in the opposite direction was observed (0/38). This asymmetry, when comparing luminal A vs. non-luminal A in the primary tumour and the lymph node, was significant (*p* = 0.02, McNemar-Bowker test of symmetry). The remaining two cases shifted from HER2-positive and triple negative in the primary tumour to luminal B subtype and HER2-positive in the lymph node (Table 2).

**Table 1:** Clinicopathological data of the included patients

**Characteristics**		**Number of patients (%)**
All patients		85 (100)
Age	Median (range)	64 (26–76)
HER2	Positive	17 (20)
	Negative	68 (80)
Ki67	≤ 20%	54 (74)
	> 20%	19 (26)
ER status	Positive	62 (73)
	Negative	23 (27)
Pr status	Positive	41 (48)
	Negative	44 (52)
Histology	Ductal	67 (81)
	Lobular	10 (12)
	Ductal + Lobular	4 (5)
	Other	2 (2)
	Missing	2
Tumor size	≤ 20%	25 (29)
	> 20%	60 (71)
Grade	1	2 (3)
	2	36 (60)
	3	22 (37)
	Missing	25
Nodal status	N1	22 (26)
	N2-3	25 (29)
	N4+	38 (45)
Events^1^	Yes	25 (29)
	No	60 (71)

*Abbreviations: ER* oestrogen receptor, *PR* progesteron receptor, *HER2* human epidermal growth factor receptor 2, *Grade* Nottingham histological grade.

^1^Events: recurrence and/or death in breast cancer disease.

**Table 2:** Distribution of St Gallen molecular subgroups in primary breast tumours and matched lymph node metastases

		**Molecular phenotype in lymph node metastases**				**Total**
		***N***				
		**Luminal A**	**Luminal B**	**HER2-positive**	**Triple negative**	
**Molecular phenotype in breast tumour**	luminal A	38 (85)	5 (11)	0	2 (4)	45 (100)
	luminal B	0	17 (100)	0	0	17 (100)
	HER2-positive	0	1 (9)	10 (91)	0	11 (100)
***N* (%)**	Triple negative	0	0	1 (8)	11 (92)	12 (100)
	Total	38	23	11	13	85 (100)

*p* = 0.06 McNemar-Bowker test of symmetry for all subclasses.

*p* = 0.02 McNemar-Bowker test of symmetry for Luminal A subclass *versus* non-Luminal A subclasses.

*N =* number of patients, percentages are given within parenthesis.

### Survival analysis

**Figure 1:**
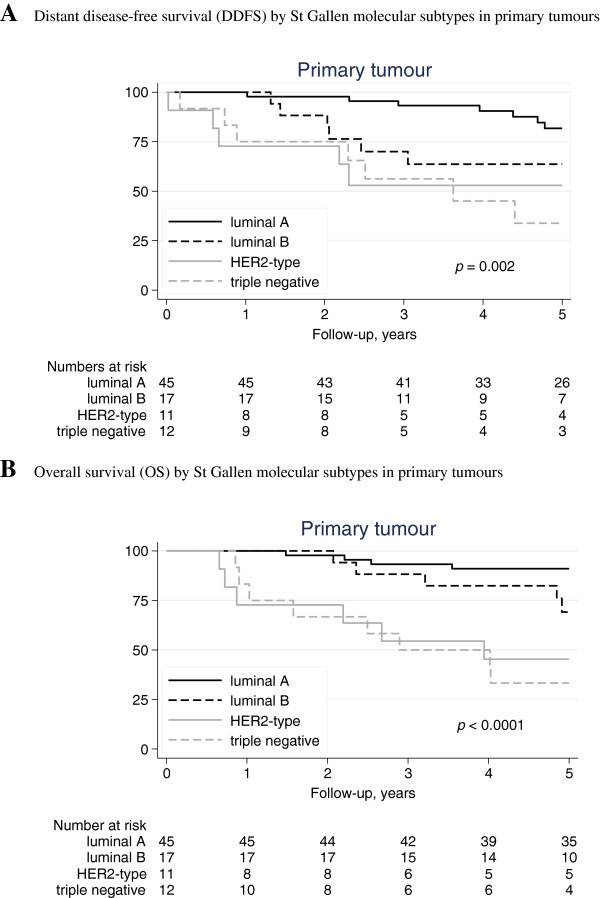
**Distant disease-free survival (DDFS) and overall survival (OS) by St Gallen molecular subtypes in primary tumours. A**. Distant disease-free survival (DDFS) by St Gallen molecular subtypes in primary tumours. **B**. Overall survival (OS) by St Gallen molecular subtypes in primary tumours.

**Figure 2:**
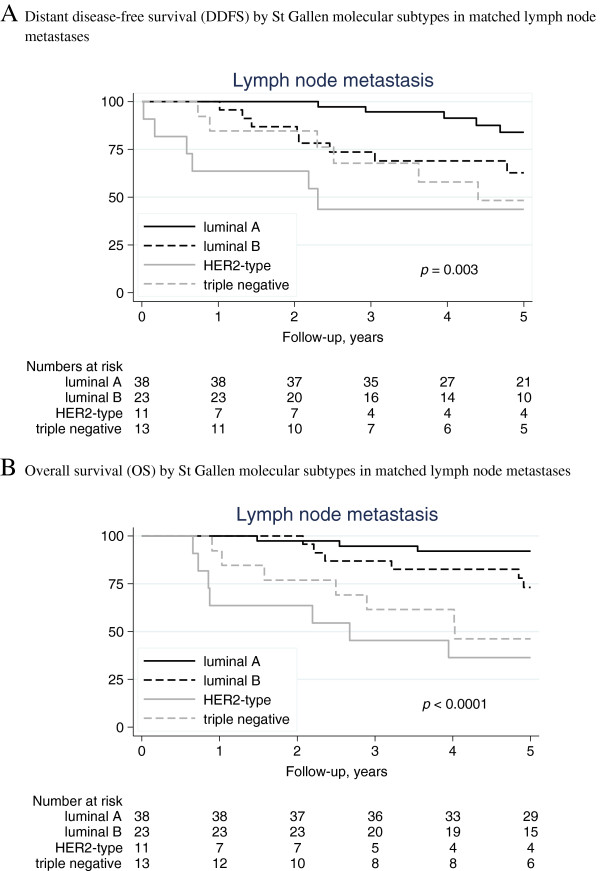
**Distant disease-free survival (DDFS) and overall survival (OS) by St Gallen molecular subtypes in matched lymph node metastases. A**. Distant disease-free survival (DDFS) by St Gallen molecular subtypes in matched lymph node metastases. **B**. Overall survival (OS) by St Gallen molecular subtypes in matched lymph node metastases.

Three degree of freedom log rank tests revealed significant differences in DDFS and OS between the subtypes for both primary tumors (*p* = 0.002 and *p <* 0.001, respectively) and lymph node metastases (*p* = 0.003 and *p <* 0.001, respectively) with the HER2-positive and triple-negative subtype associated with the shortest survival time (Figures 1 and 2). The difference in DDFS between the subtypes of both primary breast tumour and paired lymph node was further evaluated with Cox proportional hazards model. For both the primary breast tumour and paired lymph node all subgroups had an increased hazard of developing distant metastases or dying in breast cancer disease, compared with the luminal A subclass (Table 3). In multivariable analysis, adjusting for calendar-period, age at time of diagnosis and study regime (postmenopausal *versus* premenopausal cohort), results were similar although not statistically significant for all subtypes (Table 3).

**Table 3:** Cox proportional hazards regression analysis of 5-year distant disease-free survival according to St Gallen Molecular subtypes with luminal A as reference group

**A. Univariable analysis**						
	**Primary tumour**			**Lymph node metastases**		
	**Frequency** ***N*** **(%)**	**HR (95% CI)**	***p*-value**	**FrequencyN (%)**	**HR (95% CI)**	***p*-value**
**luminal A**	45 (53)	1.0		38 (45)	1.0	
**luminal B**	17 (20)	2.8 (0.94-8.4)	0.064	23 (27)	3.1(1.0-9.5)	0.048
**HER2-positive**	11 (13)	4.5 (1.4-14)	0.011	11 (13)	7.3 (2.2-24)	0.001
**Triple negative**	12 (14)	5.9 (2.1-17)	0.001	13 (15)	4.6 (1.4-15)	0.011
**B. Multivariable analysis**						
	**Primary tumour**^**1**^			**Lymph node metastases**^**1**^		
	**Frequency** ***N*** **(%)**	**HR (95% CI)**	***p*-value**	**FrequencyN (%)**	**HR (95% CI)**	***p*-value**
**luminal A**	45 (53)	1.0		38 (45)	1.0	
**luminal B**	17 (20)	2.9 (0.96-8.8)	0.059	23 (27)	2.9 (0.92-9.0)	0.070
**HER2-positive**	11 (13)	3.1 (0.94-10)	0.063	11 (13)	4.8 (1.3-17)	0.016
**Triple negative**	12 (14)	4.6 (1.6-13)	0.006	13 (15)	3.9 (1.2-13)	0.029

^1^Adjusted for calendar-period (year at time of operation), age (years) and study regime (postmenopausal versus premenopausal study).

*Abbreviations*: *HER2* human epidermal growth factor receptor 2, *HR* hazard ratio, *CI* confidence interval, *N* number of patients, percentages are given within parenthesis.

Patients switching from luminal A in the primary tumour to non-luminal A in the lymph node metastases (n = 7) had no significant change in prognosis compared to the stable luminal A subgroup (n = 38) or to the stable non-luminal A subgroup (n = 40) in terms of DDFS and OS (data not shown). However, the number of patients shifting from luminal A to non-luminal A are few, and no definitive conclusions can be drawn from this study. One patient shifting from triple-negative in the primary tumour to HER2-positive subtype in the lymph node metastases had distant metastases and died within one year, whereas the patient shifting from HER2-positive subtype to a luminal B subtype was without any event at 5 years follow-up.

## Discussion

Combining biological tumour markers into surrogate molecular subtypes has been shown to add prognostic information [6-8,10,11] which may be of importance for recommendation of systemic therapy. Unlike the analyses of individual biomarkers in the present cohort of patients [17] which showed high concordance between primary tumours and corresponding lymph node metastases, the molecular subtypes identify a subgroup of patients with ER positive disease as luminal B, with a worse prognosis, who may benefit from adjuvant chemotherapy alongside endocrine treatment [10,14,20]. We found the prognosis according to the molecular subtypes to be superior for the luminal A subtype in primary tumours as well as in synchronous lymph node metastases. A subset of patients shifting from a luminal A subtype in the primary tumour to a non-luminal A subtype in the metastatic lymph node can constitute a subgroup where adjuvant chemotherapy would have improved prognosis.

The present cohort, with patients included in two prospective trials of adjuvant tamoxifen, was initiated decades ago. The distribution of molecular subtypes in the primary tumour is, however, similar to today’s distribution with 13% of the tumours being HER2 overexpressed and more than 50% having a luminal A phenotype [11]. The finding of a shift in molecular subtype from the primary tumour to the metastases is thus not necessarily influenced by the draw-backs of including a cohort not offered modern treatment. The prognosis, however, is dependent not only on the phenotype of the tumour and the metastases, but also on the calendar-period including the treatment offered at that time. Survival analyses were adjusted also for calendar-period with similar results. The study only includes 85 patients and is not powered to find any difference in presentation of four molecular subtypes in the primary tumour *versus* metastases. Hence, the shift of a molecular subtype towards a more aggressive subtype in the metastatic lymph node is a hypothesis-generating finding in line with recent publications [21]. In the recently published study from our group [22] comparison of molecular subtypes in primary tumour and synchronous lymph node metastases also revealed a shift in individual patients. The shift was observed from luminal A to non-luminal A in the metastatic node as well as the reversed shift, from non-luminal A to luminal A in the metastatic node. In the present study, only shifts to a molecular subtype with worse prognosis were observed. The number of patients in the present study cohort is limited (N = 85) and the inclusion was restricted to patients with stage II breast cancer whom all received adjuvant treatment with tamoxifen irrespective of expression of ER as opposed to the patients in the more recent study [22] which constitutes an unselected cohort where patients were offered adjuvant treatment according to modern guidelines. The analyses of HER2 also differ between the studies, where assessment according to immunohistochemistry (IHC) (present) or silver *in situ* hybridization (SISH) [22] could affect the results. Interestingly, shifts are observed in individual patients in both patient cohorts according to molecular subtypes, proposing a molecular event in the metastatic niche during tumour cell progression with influence on prognosis.

### Tissue analysis

The individual biomarker discordance between primary tumours and metastases may reflect tumour progression, although test artefacts have also been proposed. For HER2 analysis, a recent meta-analysis including 26 primary publications has suggested that limitations of test reproducibility are less likely to explain the discordance in HER2 status found between primary tumours and metastatic sites [23]. The authors found a low HER2 discordant proportion for synchronous lymph node metastases compared to metachronous distant metastasis, supporting that tumour progression plays a major role. In the present study, biopsies were obtained by a manual arrayer from lymph nodes corresponding to the primary tumour and further processed as described previously for analyses of HER2 and Ki67. The method has limitations because a small area of one of the metastatic lymph nodes is examined. Sampling may therefore contribute to bias in representative areas of evaluation.

In the present study, 8/85 tumours were classified as HER2 2+ according to IHC analyses. In a national survey performed by our group, 12% of HER2 2+ tumours were amplified according to fluorescence *in situ* hybridization FISH [24] and in another study [25] the concordance was up to 24%. This would result in 1–2 patients of HER2 2+ tumours as amplified in the present cohort, thus patients with HER2 2+ tumours were included as HER2-negative.

### Cut-off values

The previously defined cut-off values for biomarker expression are based on accepted guidelines [26,27] in which Ki67 is the least studied with few validated guidelines available. In the present study, representative areas for the TMAs were examined to identify cancerous regions within a tissue sample. Areas in the region with increased number of Ki67 positive cells, hot spots, were identified and the number of positive cells was assessed and index calculated. The present study used a predefined 20% cut-off point based on the population sectioning, distinguishing the one third of the patients in the population with the highest proliferation from the remaining two thirds [28,29]. The prognostic value of Ki67 has been investigated in several recent publications [6,28,30] but the assessment of the cut-off value of Ki67 is not settled and the reliability of the measures varies in different geographic settings [10]. The cut-off value of ER responsiveness in clinical practice is traditionally 10%. This cut-point was chosen also in the present study, although there is support for a lower cut-off value of 1% for endocrine treatment and thus the detection of any ER positive cell in the tumour will define it as an ER responsive tumour [10]. ASCO/PAP guidelines support the 1% cut-off [27] but the guidelines are questioned in a recent study [31].

The results in this study indicate tumour instability in clinically used markers in combination classified according to the St Gallen molecular subtypes between primary breast cancer and synchronous matched lymph node metastases. Furthermore, the survival analyses show that the St. Gallen molecular subtypes have similar prognostic implications in primary tumours and matched lymph node metastases. Node status is still a powerful prognostic factor in primary breast cancer despite advanced molecular techniques. A shift in molecular characteristics to a more aggressive phenotype in synchronous nodal metastases compared to the primary tumour suggests that tumour progression occurs already at time of diagnosis in a fraction of breast cancer patients with node positive disease. The selection of more aggressive cell clones in lymph node metastases can be an additional explanation to the prognostic information gained by nodal involvement in primary breast cancer, besides a more advanced stage of the disease.

## Conclusions

The present study shows that a proportion of the ER positive group of patients with a luminal A subtype in the primary tumour gain proliferation and/or HER2 amplification in the metastatic lymph node and switch inherence to a subtype with impaired DDFS. Data from patients with metastatic breast cancer suggests that selection of systemic therapy should be guided by biomarker analysis in the metastases [13,32]. If adjuvant treatment selection is to be based also on the molecular subtype in synchronous lymph node metastases, chemotherapy would have been advocated for patients shifting from luminal A to a non-luminal A subtype, alongside endocrine treatment. Biomarker analysis in matched lymph node metastases could easily be implemented in clinical practice if it would be of value for adjuvant treatment selection. Future studies including larger cohorts of patients are necessary in order to evaluate the results of the present study before they can be translated into clinical practice.

## Methods

### Patients

**Figure 3:**
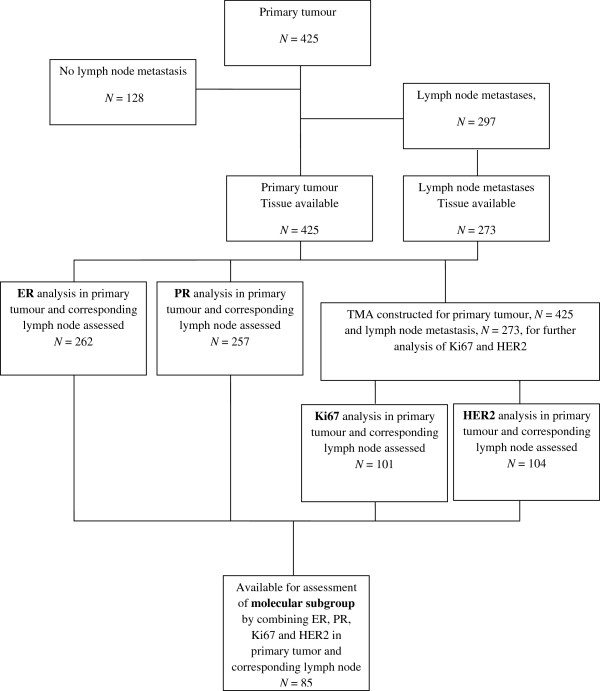
**Flow chart of study cohort.** Abbreviations: ER = Oestrogen receptor, PR = Progesterone receptor, HER2 = Human epidermal growth factor receptor 2, TMA = Tissue microarray.

The study is based on a cohort of patients previously selected from two prospective randomised clinical trials to investigate the compatibility of different laboratory methods for the evaluation of hormonal receptor status [33]. The original studies included patients from the South–Swedish Health Care Region (hospitals in Simrishamn, Ystad, Trelleborg, Malmö, Lund, Landskrona, Hässleholm, Ängelholm, Kristianstad, Halmstad, Ljungby, Växjö, Karlskrona and Karslhamn) during 1985–1994 irrespective of hormonal receptor status and with stage II unifocal, radically operated early breast cancer without distant metastases. In the postmenopausal study, the patients were allocated to 2 years (n = 496) versus 5 years (n = 469) of adjuvant tamoxifen treatment [34]. For premenopausal patients, identical inclusion- and exclusion criteria were used except for menopausal status and patients were allocated to two years of tamoxifen (n = 213) versus no adjuvant treatment (n = 214) [35]. No other adjuvant therapy was allowed and less than 1% of the premenopausal patients received polychemotherapy. The original cohort of the quality-assurance study included 425 patients treated with adjuvant tamoxifen for two years, 297 of whom had lymph node metastases (Figure 3). All the patients underwent surgical treatment of the breast and axilla. Radiotherapy was given to the breast in the case of breast-conserving surgery, and locoregionally if lymph node metastases were present. Adjuvant systemic treatment was given as 2 years of tamoxifen irrespective of hormone receptor status. The patients had annual mammograms and physical investigations for 5 years. For the patients who were classified in the present study (n = 85), the median follow-up for DDFS was 5.1 years for patients alive and without metastases. The trial was approved by the Ethics committee at Lund University (LU240-01) and informed consent was obtained from all included patients.

The cohort of patients was recently re-examined for differences in individual biomarker presence between the primary tumour and the lymph node metastases [17]. Information on clinical outcome as well as patient and tumour characteristics was therefore already available. In the present study, it was possible to classify 85 patients from the original cohort into the four subtypes of luminal A, luminal B, HER2-positive and triple negative according to ER, PR, HER2 and Ki67. The immunohistochemical staining of primary tumours and lymph node metastases was performed at the same time. Patient and tumour characteristics for these 85 patients are summarised in Table 1. They reveal a cohort of patients with known metastases in the axilla, so the fraction of events is, as expected, high (25/85). This is also reflected by the high fraction of large tumours (>20 mm: 71%).

### Tissue microarrays

In the previous study [17], tissue microarrays from primary tumours and ipsilateral lymph node metastases were constructed for analysis of Ki67 and HER2. Representative areas of invasive breast cancer, embedded in paraffin blocks, were marked. Two cores (0.6 mm) from each tumour block of the primary tumour were punched out and one biopsy specimen from the corresponding lymph node metastases was obtained by a manual arrayer (Beecher Instruments, Sun Prairie, WI, USA) and positioned into a recipient paraffin array block. Staining with haematoxylin and cytokeratin (AE1/AE3) was carried out for a morphological overview and the localization of cancer cells. One section per tissue specimen (primary tumour and lymph node metastases) and biomarker was evaluated.

HER2 scoring was determined after staining with a primary antibody (A0485, DAKO, Glostrup, Denmark) using a standard protocol (HercepTest™) to quantify and categorize tumours into four groups: 0: no staining in all tumour cells, or membrane staining in fewer than 10% of tumour cells; grade 1+: weak, not circumferential staining in more than 10% of the tumour cells; grade 2+: intermediate, circumferential staining in more than 10% of the tumour cells; and grade 3+: intense and circumferential membrane staining in more than 10% of the tumour cells. HER2 scoring was denoted as HER2-positive for all 3+ tumours and HER2-negative in 0, 1+ and 2+.

The Ki67 labelling index was determined using the antibody MIB-1 (M7240, DAKO). Sections of 4 μm were cut, mounted onto capillary microscope slides (DAKO), dried overnight at room temperature followed by 1–2 h at 60°C. The sections were deparaffinized in xylene and rehydrated in a graded series of ethanol. Antigen retrieval was performed in a microwave oven, pH 9 buffer (S2367, DAKO). Staining was performed using an automatic immunostainer (TechMate™ 500 Plus, DAKO) with an incubation time of 30 min at room temperature and with MIB-1 diluted 1:1000. DAKO Envision™ (DAKO,) was used as the visualization system. Diaminobenzidene was used as the chromogen. The IHC staining was examined by light microscopy by two independent observers. A cut-off point of > 20% labelled nuclei was used to demarcate high Ki67 [28,29].

ER and PR were previously analyzed with IHC on formalin-fixed, paraffin-embedded breast carcinoma on whole slides, and were considered positive when more than 10% of the nuclei were stained [33].

### Molecular subtype classification

The categorisation of molecular subtypes was constructed according to the St Gallen International Breast Cancer Conference 2011 [10]: luminal A (ER + and/or PR+, Ki67 low and HER2-), luminal B (ER + and/or PR+, Ki67 high and/or HER2+), HER2-positive (ER-, PR- and HER2+) and triple-negative type (ER-, PR- and HER2-). In 85 patients of 297 with lymph node metastases, all markers were known and the patients were possible to classify into the four subtypes.

### Statistical methods

The classifications of primary breast cancer tumours and corresponding lymph node metastases by molecular subtypes were compared using the exact McNemar-Bowker test of symmetry. The null hypothesis of this test is that the matrix formed by cross tabulation of the molecular subtype variables is symmetric and the alternative that it is not. Significant deviation from symmetry indicates a non-random subtype shift from the primary tumour to the lymph node metastases. The test is a generalisation of the McNemar test to more than two categories. Distant disease-free survival (DDFS) was the primary end-point and included any distant relapse (lung, liver, bone, brain, bowel) or breast cancer death as primary event and was calculated from the day of operation until the first event or the last review of the patient’s record. Overall survival was the secondary endpoint and included deaths of any cause. The molecular subtypes were related to clinical outcome in terms of DDFS by Cox analysis with luminal A as the reference group. Proportional hazards assumptions were checked with Schoenfeld’s test and deviations from proportionality were observed for the nominal molecular subtype variables (3-df tests). To reduce this problem, the follow-up was restricted to the first 5 years after diagnosis, but also for this interval, the hazard ratios should be interpreted as time average effects because the effects level off with time.

P-values less than 0.05 were considered significant. The statistical software package Stata 12.1 (Stata Corp. College Station, TX, USA) was used for all the statistical calculations.

## Abbreviations

ER: Oestrogen receptor; PR: Progesterone receptor; HER2: Human epidermal growth factor receptor 2; NHG: Nottingham histological grade; DDFS: Distant disease-free survival; OS: Overall survival; IHC: Immunohistochemistry; ISH: *in situ* hybridization; HR: Hazard ratio; CI: Confidence interval.

## Competing interests

The authors declare that they have no competing interests.

## Authors’ contributions

AKF was responsible for data acquisition, participated in the statistical analyses and drafted the manuscript. POB was responsible for database coordination and statistical analyses. MF was participating in initiation and design of the study together with LR who also was responsible for data acquisition, participating in statistical analyses and drafting of the manuscript. All authors read and approved the final version of the manuscript.

## Pre-publication history

The pre-publication history for this paper can be accessed here:

http://www.biomedcentral.com/1471-2407/13/558/prepub
